# Involvement of a Novel Genistein-Inducible Multidrug Efflux Pump of *Bradyrhizobium japonicum* Early in the Interaction with *Glycine max* (L.) Merr

**DOI:** 10.1264/jsme2.ME13057

**Published:** 2013-11-13

**Authors:** Keisuke Takeshima, Tatsuo Hidaka, Min Wei, Tadashi Yokoyama, Kiwamu Minamisawa, Hisayuki Mitsui, Manabu Itakura, Takakazu Kaneko, Satoshi Tabata, Kazuhiko Saeki, Hirofumi Oomori, Shigeyuki Tajima, Toshiki Uchiumi, Mikiko Abe, Yoshihiko Tokuji, Takuji Ohwada

**Affiliations:** 1 Department of Agricultural and Life Sciences, Obihiro University of Agriculture and Veterinary Medicine, Inada-cho, Nishi 2–11, Obihiro, Hokkaido, 080–8555, Japan; 2 School of Life Science, Lanzhou University, 222 Tianshui South Rd, Gansu, Lanzhou, 730000, China; 3 Graduate School of Agriculture, Tokyo University of Agriculture and Technology, 3–8–1, Harumi-cho, Fuchu, Tokyo, 183–8538, Japan; 4 Graduate School of Life Science, Tohoku University, 2–1–1, Katahira, Aoba-ku, Sendai, Miyagi, 980–8577, Japan; 5 Faculty of Engineering, Kyoto Sangyo University, Kitaku, Kyoto, 603–8555, Japan; 6 Kazusa DNA Research Institute, 2–6–7, Kazusa-kamatari, Kisarazu, Chiba, 292–0818, Japan; 7 Department of Biological Science, Faculty of Science, Nara Women’s University, Kitauoyanishi-machi, Nara, 630–8506, Japan; 8 Graduate School of Science, Osaka University, 1–1, Machikaneyama, Toyonaka, 560–0043, Osaka, Japan; 9 Department of Life Science, Kagawa University, 2393 Ikenobe, Miki-cho, Kita-gun, Kagawa, 761–0795, Japan; 10 Graduate School of Science and Engineering, Kagoshima University, 1–21–24, Korimoto, Kagoshima, 890–0065, Japan

**Keywords:** *Bradyrhizobium*, genistein, multidrug efflux pump, TetR family transcriptional regulator

## Abstract

The early molecular dialogue between soybean and the bacterium *Bradyrhizobium japonicum* is crucial for triggering their symbiotic interaction. Here we found a single large genomic locus that is widely separated from the symbiosis island and was conspicuously induced within minutes after the addition of genistein. This locus (named BjG30) contains genes for the multidrug efflux pump, TetR family transcriptional regulator, and polyhydroxybutyrate (PHB) metabolism. The induction of BjG30 by genistein was competitively inhibited by daidzein, although both genistein and daidzein are soybean-derived inducers of nodulation (*nod*) genes. Such a differential expression pattern is also observed in some legume-derived flavonoids, which structurally differ in the hydroxy/deoxy group at the 5-position. In addition, not only did the induction start far in advance of *nodW* and *nodD1* after the addition of genistein, but the levels showed distinct concentration dependence, indicating that the induction pattern of BjG30 is completely different from that of *nod* genes. The deletion of genes encoding either the multidrug efflux pump or PHB metabolism, especially the former, resulted in defective nodulation performance and nitrogen-fixing capability. Taken together, these results indicate that BjG30, and especially its multidrug efflux pump, may play a key role in the early stage of symbiosis by balancing the dual functions of genistein as both a *nod* gene inducer and toxicant.

Root nodule bacteria live symbiotically with a legume and are known as Gram-negative soil microorganisms fixing molecular nitrogen to ammonia. It is reported that flavonoids such as isoflavone, flavone, and flavanone, which are released from the seeds and roots of legumes, induce the nodulation (*nod*) genes within the symbiosis island in root nodule bacteria (14, 22, 39). The Nod factor produced by *nod* genes then guides nodule formation (22). The platform of the Nod factor is synthesized by *nodABC*, which is common to the symbiosis island, and the side chain is modified by a species-specific *nod* gene product (21, 34, 43). For *Bradyrhizobium*, flavonoid is recognized by NodD (a LysR family transcriptional regulator) and NodWV (a two-component system response regulator), and induces the downstream of *nod* genes (7, 28). NwsAB (a two-component system response regulator) was found as a new regulator protein involved in the induction of *nod* genes (15, 24). Flavonoid is composed of flavan, and over 4,000 types of flavonoids have been reported in legumes (11). However, the interaction between flavonoids and the NodD of rhizobia determines the type of nodule, *i.e.*, the indeterminate type formed by *Sinorhizobium* in response to flavone and flavanone, and the determinate type by *Bradyrhizobium* in response to an isoflavone. Genistein and daidzein are major isoflavones in soybean that induce *nod* genes. In particular, genistein is known to be an inducer of all *nod* box-associated genes (19, 24, 34).

However, it was reported that flavonoids are also recognized by the TetR family transcriptional regulator, which is often encoded adjacent to the RND family efflux pump, and discharged as antimicrobial compounds produced by plants (26). The flavonoid-responsive RND family efflux pump is reported as AcrAB for *Erwinia amylovora*, ifeAB for *Agrabacterium tumefaciens*, and MexAB-OprM for *Pseudomonas syringae*. The mutation of this efflux pump resulted in the loss of antimicrobial resistance and pathogenicity of these bacteria in the host plant (3, 30, 45).

For root nodule bacteria, it was reported that flavonoids released from alfalfa seeds increased the growth rate of *Rhizobium meliloti* in a defined minimal medium (17). However, the rmrAB of *Rhizobium etli* CFN42 was found to be a multidrug efflux pump involved in tolerance to flavonoids, and mutation of this pump resulted in the reduction of nodulation ability (13). In addition, the lack of SmeAB of the *Sinorhizobium meliloti* 1021 multidrug efflux pump was reported to lead to a decrease in the competing ability of nodulation (10). Twenty-four RND family efflux pumps are encoded in the *Bradyrhizobium japonicum* genome, and its FreCAB (encoded by genes bll4319–bll4321) was found among the 101 genes that responded to genistein 8 h after treatment (24, 27, 48). It was reported that the FrrA (encoded by a gene blr4322) of TetR family transcriptional regulator was adjacent to FreCAB, and that the joint product of FrrA and genistein might control the expression of FreCAB (48). However, the role of FreCAB in symbiosis is unknown.

Our previous study (46) showed that one expression cluster, including the common *nod* gene operon within the symbiosis island, was identified 6 h after treatment with genistein, and this induction occurred in advance of a gene cluster encoding the type III secretion system (*tts*) (23). Interestingly, in the same study (46) another large expression locus was found outside the symbiosis island (7.73–7.75 Mb). This locus includes genes for the RND family efflux pump (bll7019, bll7020, bll7021), the TetR family transcriptional regulator (blr7023, bll7024), and polyhydroxybutyrate (PHB) metabolism (acetoacetate decarboxylase: blr7028; β-hydroxybutyrate dehydrogenase: blr7029). These genes were collectively and markedly induced by genistein treatment at 30 min, occurring earlier than *nod* genes. Thus, in the present study we specifically investigated the expression profile of genes in this locus and evaluated the role of the RND family efflux pump and PHB metabolism in nodulation and nitrogen fixation with *Glycine max* by constructing deletion mutants. The results demonstrated that this locus, especially the RND family efflux pump, plays a significant role in the nitrogen-fixing ability of nodules.

## Materials and Methods

### Bacterial strains and culture conditions

Bacterial strains and plasmids used in this study are shown in Table 1. The *Bradyrhizobium japonicum* strains were maintained in yeast extract-mannitol broth (YMB) (20) at 30°C, and the *Escherichia coli* strain was maintained in Luria-Bertani (LB) medium at 37°C. Arabinose-gluconate (AG) medium and HM salt medium supplemented with 0.1% (w/v) arabinose (5, 36) were used for triparental mating. Media were supplemented with appropriate antibiotics at the following concentrations (per mL) as required: for *B. japonicum*, chloramphenicol (30 μg) and streptomycin (50 μg);

### RNA isolation, hybridization and macroarray analyses

For RNA isolation, cells were pre-cultured in 20 ml YMB with appropriate antibiotics described above for 3 days and scaled up to 200 ml YMB for growth until the log-phase (OD_600_ = 0.4) at 30°C. Then, the cells were diluted with fresh YMB to 0.1 OD_600_ for the induction with genistein (5 μM) at 30°C for 30 min. RNA isolation, hybridization, and data analyses were performed as described previously (46). Duplicate array membranes, which were composed of 3,960 clones covering the whole genome of *B. japonicum* USDA110, were used in each experiment and at least two independent experiments were conducted.

### Quantitative RT-PCR

Total RNA isolation was performed as described above and the primers were designed by Primer 3 (35). Oligonucleotides of the primers used in this experiment are shown in Table 1, except for *nodW*, *nodD1* and *sigA* described previously (46, 47). Total RNA (100 ng) was used as a template and the quantitative RT-PCR reactions were carried out by MiniOpticon™ (version 3.1; Bio-Rad, Hercules, CA, USA) in combination with the QuantiTect SYBR Green RT-PCR kit (Qiagen GmbH, Hilden, Germany). Quantification was performed using the method according to the application guide provided by Bio-Rad. The housekeeping gene of *B. japonicum*, *sigA* (bll7349), was used as an internal reference as mentioned previously (46).

### Construction of *B. japonicum* USDA110 mutants

**Fig. 1:**
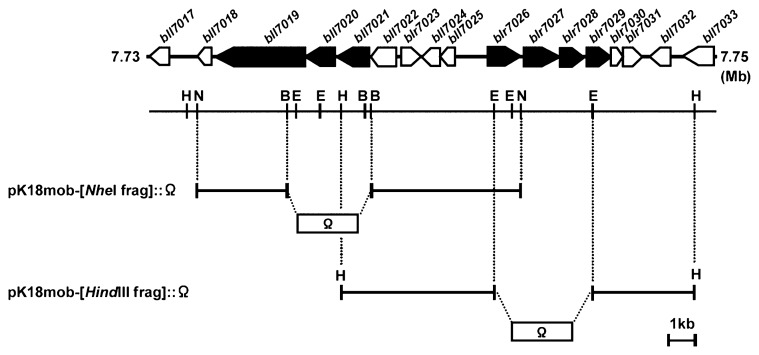
Genetic and restriction maps of BjG30 covering genes from bll7017 to bll7033 used to construct *B. japonicum* USDA110 mutants. The recombinant plasmids for the deletion mutation from bll7019 to bll7021 (pK18mob-[*Nhe*I frag]::Ω) and from blr7026 to blr7029 (pK18mob-[*Hind*III frag]::Ω) were constructed as described in Materials and Methods. B, *Bsi*WI; E, *Eco*RV; H, *Hind*III; N, *Nhe*I. Gene annotation (Rhizobase, http://www.kazusa.or.jp/rhizobase/): bll7017, LuxR family transcriptional regulator; bll7018, unknown protein; bll7019, AcrB/AcrD/AcrF family protein; bll7020, efflux protein; bll7021, HlyD family secretion protein; bll7022, unknown protein; blr7023 and bll7024, TetR family transcriptional regulators; bll7025, hypothetical protein; blr7026, unknown protein; blr7027, hypothetical protein; blr7028, acetoacetate decarboxylase; blr7029, β-hydroxybutyrate dehydrogenase; blr7030, unknown protein; blr7031, hypothetical protein; bll7032, putative cyclic NTP-binding protein; bll7033, LuxA-like protein.

Recombinant plasmid for the deletion mutation from bll7019 to bll7021 of *B. japonicum* USDA110 was constructed as follows: the *Nhe*I fragment including bll7019 to bll7021 of *B. japonicum* USDA110 (11.1 kb) was isolated from brc02044 clone of this strain and ligated into a mobilizable suicide vector, pK18mob, at the site of *Xba*I to create pK18mob-[*Nhe*I frag]. The Ω cassette, which encodes resistance to spectinomycin and streptomycin, was digested with *Sma*I from pHP45Ω and ligated into the *Bsi*WI site of pK18mob-[*Nhe*I frag] to create pK18mob-[*Nhe*I frag]::Ω (Fig. 1). For recombinant plasmid for the deletion mutation from blr7026 to blr7029, the *Hind*III fragment including blr7026 to blr7029 of *B. japonicum* USDA110 (12.2 kb) was isolated from brc00911 clone of this strain and ligated into the pK18mob at the site of *Hind*III to create pK18mob-[*Hind*III frag]. The Ω cassette was digested with *Sma*I from pHP45Ω and ligated into the *Eco*RV site of pK18mob-[*Hind*III frag] to create pK18mob-[*Hind*III frag]::Ω (Fig. 1). Then, the recombinant plasmid was introduced into *B. japonicum* USDA110 by triparental mating using pRK2013 as a helper plasmid according to Simon *et al.* (12, 38). Mutated strains were selected on AG agar plates with chloramphenicol and streptomycin, and double crossover mutants (*i.e.*, deletion mutant from bll7019 to bll7021 designated as ΔRND mutant, and deletion mutant from blr7026 to blr7029 as ΔPHB mutant) were confirmed by both kanamycin sensitivity and PCR using primers designed within the deleted gene and on the flank of the Ω-inserted site as described by Sugawara *et al.* (41).

### Plant growth, inoculation, and acetylene reduction activity

The *B. japonicum* cells were grown in YMB supplemented with the appropriate antibiotics. After centrifugation at 8,000 rpm for 5 min, the collected cells were washed three times with sterilized phosphate-buffered saline (PBS) and the cell density was adjusted to 10^7^ cells per ml with PBS. Seeds of *Glycine max* L. cv. Enrei were purchased from Kaneko Seeds Co., Ltd. (Gunma, Japan). The seeds were surface sterilized with 70% (v/v) ethanol for 1 min, 5% sodium hypochlorite for 2 min in this order, and then rinsed sufficiently with sterilized distilled water. Sterilized seeds were placed in a seed bag with Norris & Date medium (9) and inoculated with 10^7^ cells per seed. The plants were incubated under light (23.5°C for 14 h) and dark (20°C for 10 h) conditions, respectively. The number, weight and acetylene reduction activity (ARA) of nodules formed in the plant root were determined at 40 days after inoculation.

The nitrogen-fixing ability of the nodules was measured by the ARA as follows: the whole plants were sealed in a container containing 10% acetylene and incubated at 30°C for 30 min. A portion of the gas from the sealed container was withdrawn and subjected to gas chromatography (Shimadzu GC-8A, Kyoto, Japan) equipped with a column of Porapak N (50/80 mesh; Waters, Milford, MA01757, U.S.A).

## Results

### One large genistein-induced locus outside the symbiosis island

**Fig. 2:**
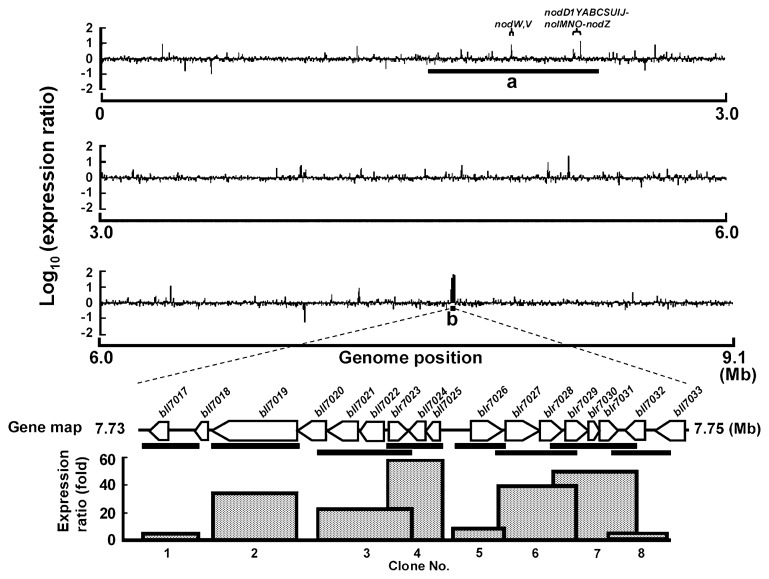
Expression profiles of whole genome and clones in BjG30 covering genes from bll7017 to bll7033 of *B. japonicum* USDA110 in response to genistein at 0.5 h post-induction (hpi). Expression profiles were constructed as the value of log_10_ (expression ratio) of 3,960 clones covering the whole genome, and the relative expression levels of each clone with and without genistein were represented by a single bar. The symbiosis island (a) is located at coordinate 1.68–2.36 Mb. The expression profiles of each clone, which are shown as bars below the gene map, in a genomic locus (b) (*i.e.*, BjG30) are shown as the box chart. This locus is located at coordinate 7,729,680–7,748,456 and is composed of eight clones covering genes from bll7017 to bll7033. Coordinate of each clone: clone 1 (brb19679) 7,729,680–7,731,626; clone 2 (BJ7162) 7,732,105–7,735,128; clone 3 (brb12742) 7,735,902–7,739,183; clone 4 (brb07276) 7,738,326–7,740,920; clone 5 (brb06980) 7,740,752–7,742,620; clone 6 (brb11559) 7,742,522–7,745,552; clone 7 (brb08642) 7,744,483–7,747,504; clone 8 (brb00329) 7,746,514–7,748,456 (accessible from the Web database, http://orca10.bio.sci.osaka-u.ac.jp/array02/).

Fig. 2 shows the expression profile of the whole genome (9.1 Mb) of *B. japonicum* USDA110 at 30 min after the addition of genistein (final conc. 5 μM). The result showed that one large genomic locus (7.73–7.75 Mb), which was widely separated from the symbiosis island (1.68–2.36 Mb), was significantly induced in the presence of genistein, and the induction levels reached 3.8- to 57.4-fold. We designated this large expression locus “BjG30.” BjG30 contained eight clones covering 17 genes from bll7017 to bll7033 (at coordinates 7,729,680–7,748,456), and two loci (clone 2–4 and 6–7) were conspicuously induced to more than 21.2-fold. The former locus covered seven genes that encoded an AcrB/AcrD/AcrF family protein (bll7019), an efflux protein (bll7020), a HlyD family secretion protein (bll7021), an unknown protein (bll7022), TetR family transcriptional regulators (blr7023, bll7024), and a hypothetical protein (bll7025).

The AcrB/AcrD/AcrF family protein, efflux protein and HlyD family secretion protein (bll7019–bll7021) are thought to be involved in the RND family efflux pump (18), whereas the latter locus (clone 6–7) covers six genes that encoded the hypothetical protein (blr7027), acetoacetate decarboxylase (blr7028), β-hydroxybutyrate dehydrogenase (blr7029), an unknown/hypothetical protein (blr7030, blr7031), and putative cyclic NTP-binding protein (bll7032). It was reported that both acetoacetate decarboxylase and β-hydroxybutyrate dehydrogenase (blr7028–blr7029) were involved in the catalytic reactions from polyhydroxybutyrate (PHB) to acetone (42).

### Strong and specific induction of BjG30 by genistein

**Fig. 3:**
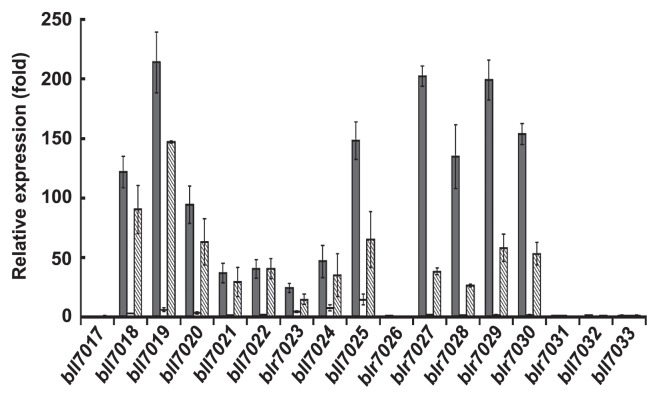
Expression profiles of BjG30 genes from bll7017 to bll7033 of *B. japonicum* USDA110 in response to genistein and/or daidzein at 0.5 hpi. The relative expression levels were normalized on the housekeeping gene of *B. japonicum*, *sigA* and represented as means ± SD of three replicates. Bars: 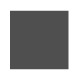, genistein (5 μM);□, daidzein (5 μM); 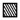, genistein (5 μM) and daidzein (5 μM).

Some adjacent clones of the macroarray used in this study overlapped, and each of the adjacent clones contained at least one gene. We thus validated the relative expression levels of all genes involved in BjG30 with quantitative RT-PCR (Fig. 3). The primers of these genes were designed as shown in Table 1. The results showed that the significant expression of all but five genes (bll7017, blr7026, blr7031, bll7032 and bll7033) was confirmed 30 min after the addition of genistein, and the induction levels were from 24.6- to 213.9-fold (Fig. 3). The levels of bll7018–bll7020, bll7025 and blr7027–blr7030 were higher than those of the other genes, indicating that the expression profile obtained by each gene corresponded well to the results of the macroarray analyses (Figs. 2 and 3).

Concomitantly, we studied the relative expression levels of these genes with daidzein (final conc. 5 μM) (Fig. 3). The results showed that the induction levels of all genes were considerably lower than those in the presence of genistein, with the maximum level (for bll7025) of at most 14.7-fold. Further, the simultaneous addition of genistein and daidzein led to an overall decrease in the expression levels of genes, with the exception of bll7022. These results suggest that BjG30 was likely induced in a genistein-specific manner and the induction levels were competitively inhibited by daidzein.

### Induction of BjG30 by 5-hydroxy/deoxy flavonoids

**Fig. 4:**
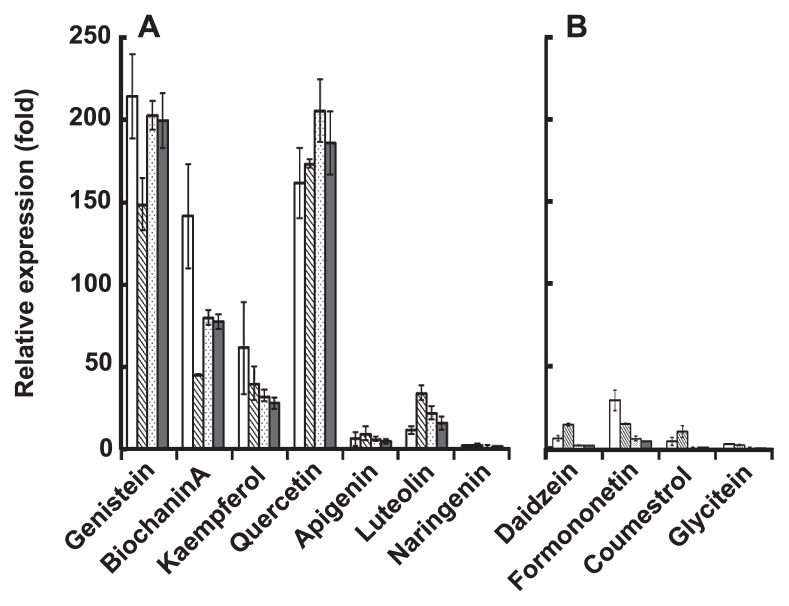
Expression profiles of BjG30 genes in response to 5-hydroxy (5 μM) (A) and 5-deoxy (5 μM) (B) flavonoids at 0.5 hpi. Four representative BjG30 genes (□, bll7019; 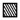, bll7025; 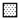, blr7027 and 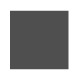, blr7029) were used. The relative expression levels were normalized on the housekeeping gene of *B. japonicum*, *sigA* and presented as the means ± SD of three replicates.

Since BjG30 was strongly induced by genistein but not by daidzein (Fig. 3), both of which are isoflavonoids with a similar molecular structure except for the hydroxy/deoxy group at the 5-position, we compared the induction levels of BjG30 between 5-hydroxyflavonoids (genistein and biochanin A as isoflavones, kaempferol and quercetin as flavonols, apigenin and luteolin as flavones, and naringenin as a flavanone) and 5-deoxyflavonoids (daidzein, formo-nonetin and glycitein as isoflavones and coumestrol as a coumestan) using four markedly induced genes (bll7019, bll7025, blr7027 and blr7029). As shown in Fig. 4, almost all of the 5-hydroxyflavonoids could induce these representative genes, although there was a difference in the induction levels. The extent of induction by quercetin was nearly the same as that by genistein, followed in descending order by biochaninA, kaempferol, luteolin and apigenin; there was no significant induction by naringenin. In contrast, the 5-deoxyflavonoids used in this experiment did not show predominant induction as a whole. These results indicate that BjG30 was significantly induced by the isoflavone, flavonol and flavone with a 5-hydroxy group.

### Effects of the concentration and treatment periods of genistein on BjG30 induction

**Fig. 5:**
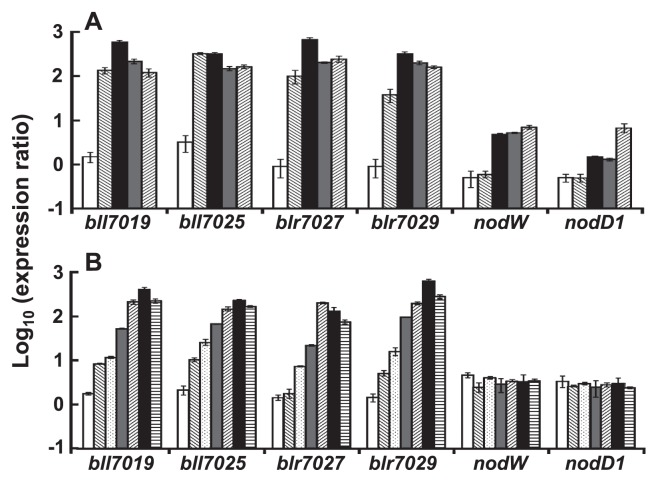
Expression patterns of BjG30 and *nod* genes of *B. japonicum* USDA110 at A, various induction periods and B, various concentrations of genistein at 30°C. Four representative BjG30 genes (bll7019, bll7025, blr7027 and blr7029) and two *nod* genes (*nodW* and *nodD1*) were used. The relative expression levels were normalized on the housekeeping gene of *B. japonicum*, *sigA* and presented as the means ± SD of three replicates. Panel A: □, 1 min; 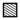, 5 min; ■, 15 min; 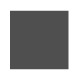, 30 min; 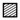, 60 min. Panel B: □, 0.1 μM; 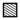, 0.5 μM; 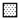, 1.0 μM; 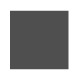, 2.5 μM; 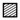, 5 μM; ■, 7.5 μM; 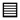, 10 μM.

We treated *B. japonicum* cells with genistein (final conc. 5 μM) at 30°C for 1–60 min and compared the induction levels of the representative genes (bll7019, bll7025, blr7027 and blr7029) with those of the nodulation genes *nodW* and *nodD1* (Fig. 5). The results showed that the induction of these BjG30 genes started at 5 min, occurring in advance of *nodW* and *nodD1*, and peaked at around 15 min. After that, the increased levels tended to decrease with time. In contrast, the induction of both *nodW* and *nodD1* occurred at 15 min and tended to increase with time. Next, we treated *B. japonicum* cells with genistein (0.1 to 10 μM) for 30 min and compared the induction levels with those of *nodW* and *nodD1*. The results showed that the induction levels of the BjG30 genes were distinctly enhanced with the increase of genistein concentration and peaked at approximately 7.5 μM. In contrast, the induction levels of these nodulation genes did not show significant changes at these concentrations. These results indicate that the induction pattern of BjG30 was completely different from that of the nodulation genes.

### Symbiotic phenotype of ΔRND and ΔPHB mutants of *B. japonicum* on soybean

Table 2 shows the number, weight and acetylene reduction activity (ARA) of nodules formed in *Glycine max* with the deletion mutants (ΔRND and ΔPHB mutants) or wild-type of *B. japonicum* at 40 days after inoculation. The results demonstrate that the nodule weight per plant inoculated with mutant strains was significantly decreased to around 75% and the weight per nodule formed with the ΔRND mutant was markedly decreased to around 54% compared to the wild type. In addition, for the ΔRND mutant, the ARA of nodules was decreased to approximately 61% of the wild type, but was not substantially changed for the ΔPHB mutant. These results indicate that the disruption of one genomic locus within BjG30 from gene bll7019 to bll7021, which are responsible for the RND family efflux pump, decreased the nodule weight and ARA, but increased the nodule number per plant.

### Growth sensitivity of ΔRND and ΔPHB mutants of *B. japonicum* against genistein or daidzein

**Fig. 6:**
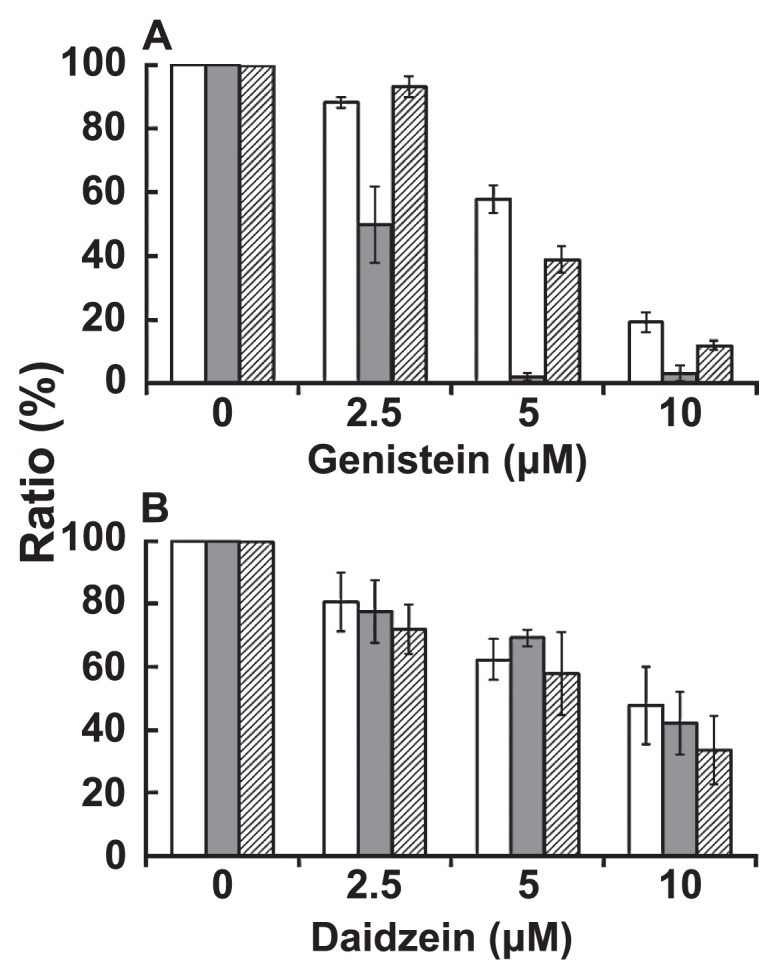
Growth of deletion mutants and wild-type *B. japonicum* USDA110 at various concentrations of genistein or daidzein. The cells were grown in YMB with genistein (0 to 10 μM) or daidzein (0 to 10 μM) at 30°C aerobically and the cell density was determined at 48 h after the incubation. Vertical axis shows the ratio (%) of cell density with genistein or daidzein to that without these flavonoids. Data are presented as the means ± SD of three replicates. Bars: □, wild type; 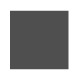, ΔRND mutant; 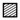, ΔPHB mutant.

Fig. 6 shows the ratio of the cell density with the addition of genistein or daidzein to that without them at 48 h. The data show that the growth of all strains fell with the elevating concentration of either genistein or daidzein, being more strongly affected by genistein. It was reported that the growth of *B. japonicum* cells was inhibited by coumestrol, genistein and daidzein, and genistein showed the highest inhibitory effect at a concentration of 20 μM (25). The percentage of the ΔRND mutant was considerably diminished compared to the wild type in the presence of genistein: 49.8% (88.1% for wild type) at 2.5 μM, 2.0% (57.9% for wild type) at 5.0 μM, and 3.2% (19.3% for wild type) at 10 μM. The percentage of the ΔPHB mutant was also decreased with the increase of genistein concentration, but the value was 11.9% even at 10 μM, indicating that the ΔRND mutant was much more sensitive to genistein than the wild type.

In contrast, in the presence of daidzein, the percentage of both mutants was 33.6% to 77.6% (47.6%–80.5% for wild type) at 2.5 to 10 μM, indicating that the sensitivity of both mutants to daidzein was more or less similar to that of the wild type. These results demonstrate that the disruption of the gene locus bll7019 to bll7021 considerably enhances the sensitivity to genistein but not daidzein, indicating that the RND family efflux pump encoded by this gene locus might play a key role in the efflux of genistein outside the cell.

## Discussion

In this study, locus BjG30 was identified as a single large genomic locus located outside the symbiosis island, strongly induced by genistein as early as 5 min. The expression profile of BjG30 induced by genistein differed completely from that of *nod* genes such as *nodD1* and *nodW* (Fig. 5), although the activation of *nodABC*-*lacZ* fusions was reported to depend on genistein concentration (22). In addition, no *nod* box motif (24) existed in this locus. Thus, we suspect that the induction of BjG30 occurred through the TetR family transcriptional regulator instead of *nodD* and *nodW*. Lang *et al.* (24) proposed a model in which the TetR family transcriptional regulator regulated gene expression by recognizing genistein. It was also reported that the TetR family transcriptional regulator of some pathogens recognized flavonoids such as genistein of plants and regulated the expression of their genes (3, 30, 45). However, there has been no prior report about a BjG30 locus which was strongly induced 5 min after genistein treatment, to the best of our knowledge.

When we inoculated soybean plants with the ΔRND mutant (inactivated RND family efflux pump), both the weight and nitrogen-fixing ability of the nodules were significantly decreased, although the nodule number per plant was increased (Table 2). The BdeAB encoded by blr1515 and blr1516 is also known as another RND family efflux pump of *Bradyrhizobium*, playing a role in the tolerance to kanamycin and gentamycin (27). Here, the destruction of BdeAB resulted in an increase in nodule number and a decrease in both the weight and nitrogen-fixing ability of the nodules. It was also proposed that the BdeAB pump participates in the growth of *B. japonicum* cells inside the plant because the number of viable cells isolated from the nodules formed by this mutant was decreased (27).

In the present study, genes (bll7019–bll7021) encoding the RND family efflux pump were significantly induced by genistein but not daidzein (Fig. 3), and the susceptibility of the ΔRND mutant to genistein was considerably enhanced compared to the wild type (Fig. 6). These results suggest that the RND family efflux pump in locus BjG30 also participates in the growth manipulation of *Bradyrhizobium* cells inside the plant, with a functional role in the specific excretion of 5-hydroxyflavonoids such as genistein, which finally results in the toxic alleviation of the cells. It was reported that 5-hydroxyflavonoids such as genistein, quercetin and kaempferol are present in soybean (31).

The DNA, RNA and protein synthesis in *Vibrio* and *Bacillus* cells were reported to be inhibited more strongly by genistein than by daidzein (44), and genistein inhibited the activity of tyrosine kinase and DNA topoisomerase II by binding to an ATP-binding site (1, 29). In particular, the activity of DNA topoisomerase II was inhibited by 5-hydroxyflavonoids such as genistein, biochanin A, quercetin and kaempferol, but not by daidzein (1, 2, 6, 29). Since the RND family efflux pump in BjG30 was induced mainly by these flavonoids, we suspect that this efflux pump can discharge mainly 5-hydroxyflavonoids such as genistein out of the cells so that *B. japonicum* cells can grow in such a way as to prevent the inhibition of DNA topoisomerase II.

However, when the ΔPHB mutant (inactivated acetone production from PHB) was inoculated into soybean, the nodule weight per plant was significantly decreased, although the number and nitrogen-fixing ability of the nodules were not significantly different from those of the wild type (Table 2). Since both acetoacetate decarboxylase and β-hydroxybutyrate dehydrogenase are involved in the catalytic reactions from PHB to acetone, it is possible that the production of acetone is induced by genistein, and lack of this production results in decreased nodule weight. Suganuma *et al.* reported that acetone and acetoacetate decarboxylase were produced in nodules formed with the combination of *Bradyrhizobium* and *Glycine max*, but the role of acetone in symbiosis is still unknown (40). Between the two genes (blr7030 and blr7031) located downstream of the target genes (blr7028 and blr7029) for ΔPHB mutant, only blr7030 was induced to approximately 153.8-fold in the presence of genistein (Fig. 3). However, the genistein-inducible expression of blr7030 did not occur in ΔPHB mutant (data not shown). It is possible that a lack of blr7030 expression affects the nodulation performance of ΔPHB mutant, although the function of this gene product is unknown. The effect of blr7030 product on symbiosis is under investigation.

Gene replacement and double crossover mutants were verified by antibiotic (*i.e.*, kanamycin) sensitivity and by PCR using primers for the deleted genes and on the flank of the Ω-inserted site according to Sugawara *et al.* (41), as described in Materials and Methods. In addition, there is no homologous DNA region with DNA on the flank of the Ω-inserted site in the whole genome (based on Rhizobase, http://www.kazusa.or.jp/rhizobase/), indicating that only the target locus was genetically modified. For the complementation test, the brc02044 cosmid clone (at coordinate 7,723,981 to 7,747,611) containing a DNA region from bll7019 to bll7021 and the brc00911 cosmid clone (at coordinate 7,735,146 to 7,758,873) containing from blr7026 to blr7029 were introduced into the ΔRND and ΔPHB mutants, respectively. However, the defective nodulation performance was not complemented (data not shown). Since an inoculation test ought to be conducted in the absence of antibiotics to avoid the influence on plant growth, these results seem to suggest that the cosmid clone was not properly maintained in these mutants. Further investigation is being conducted.

It is generally known that protons are consumed in the decarboxylation reaction (16). For *Escherichia coli*, it was reported that intracellular pH was regulated by the consumption of protons in the decarboxylation reaction (4, 8). In contrast, it is reported that the RND family efflux pump takes protons into the cell while discharging drugs out of the cell (32). Thus, intracellular pH might be regulated by the consumption of protons, which are taken into the cell while discharging toxicants such as genistein, in the decarboxylase reaction catalyzed by acetoacetate decarboxylase.

The results of the present study show that a single large genomic locus containing genes for the multidrug efflux pump and PHB metabolism outside the symbiosis island was rapidly and mainly induced after treatment with 5-hydroxyflavonoids such as genistein. Although some 5-hydroxyflavonoids such as quercetin did not induce the *nod* genes in *B. japonicum* (22), our data indicate that flavonoids, especially genistein, are likely a double-edged sword (*i.e.*, as both toxicants and *nod* gene inducers) for *B. japonicum* cells, and that *B. japonicum* has developed an elaborate genomic locus (*i.e.*, the efflux system described in this study) to balance the dual effects of flavonoids, which are important for successful symbiosis.

## Figures and Tables

**Table 1:** Bacterial strains, plasmids and oligonucleotides used in this study

Strain or plasmid	Characteristics or sequence	Source or reference
*Bradyrhizobium japonicum* strains		
USDA110	Wild-type strain	USDA, Beltsville, MD
ΔRND mutant	Deletion of *bll7019-bll7021* of USDA110/insertion of Ω; Sm^r^, Sp^r^	This study
ΔPHB mutant	Deletion of *blr7026-blr7029* of USDA110/insertion of Ω; Sm^r^, Sp^r^	This study
*Escherichia coli*		
JM109	*recA*, cloning strain	49
Plasmids^a^		
pK18mob	Mobilizable suicide vector; Km^r^	37
pHP45Ω	Plasmid carrying Ω cassette; Sm^r^, Sp^r^	33
pRK2013	ColE1 replicon carrying RK2 transfer genes; Km^r^	12
pK18mob-[*Nhe*I frag]::Ω	pK18mob carrying Ω-inserted 11.1 kb *Nhe*I fragment from brc02044 clone of USDA110	46, This study
pK18mob-[*Hind*III frag]::Ω	pK18mob carrying Ω-inserted 12.2 kb *Hind*III fragment from brc00911 clone of USDA110	46, This study
Oligonucleotides^b^		
bll7017 F/R	5′-ACACCTGCTTCTACGTCAAT-3′/5′-CTGAAGCCTTCGAGGATG-3′	This study
bll7018 F/R	5′-CACATCCGTCACCTACAAC-3′/5′-GCAGTCACTGTCAGAGGAAT-3′	This study
bll7019 F/R	5′-AACATCTATGCCTTCACCAG-3′/5′-GGAGAATTCGAGATAGATCACC-3′	This study
bll7020 F/R	5′-CGTCTGGATCGTCAATCA-3′/5′-CTGCTGATCCTGTTCGATA-3′	This study
bll7021 F/R	5′-GGAACTGTGAGAGTGAAGACC-3′/5′-CCTGCCTTGATCAACACTT-3′	This study
bll7022 F/R	5′-ATGCCGCGACTTACATT-3′/5′-GACAGCTTGACCTTGATCTC-3′	This study
blr7023 F/R	5′-AAGCTCATCGAGGAATCGTT-3′/5′-CGTCGCTTCATTCAAGAGTT-3′	This study
bll7024 F/R	5′-CAATGGATGCTTACCTCAG-3′/5′-TTAGCAGGCGGATCTGTAG-3′	This study
bll7025 F/R	5′-GCTCTATCGCAGCTTCATT-3′/5′-GCAAGGTAGGTCTCGATGAT-3′	This study
blr7026 F/R	5′-CAGACATCAGCTTCGAGATA-3′/5′-AACTCCTGCTTGATCCACTT-3′	This study
blr7027 F/R	5′-GTCTACAACATCGTTCACCTG-3′/5′-GTGCTCTAGATCGAATGTGA-3′	This study
blr7028 F/R	5′-CCGCTCGTCAAGTATGAAT-3′/5′-GGAACATGCAGTGGTTGTAG-3′	This study
blr7029 F/R	5′-GTTCAAGTCAGCCTACGTC-3′/5′-GACCTCATCTCTTCTCATCTTG-3′	This study
blr7030 F/R	5′-ACTCTGCAGCATGGTATGA-3′/5′-ATGAGGAAGGAGCATTGAC-3′	This study
blr7031 F/R	5′-GCCCAGATGAAGACCTATT-3′/5′-GTCAGACTTCAGCTGCTTG-3′	This study
bll7032 F/R	5′-TGACGATGAGCAAGCTG-3′/5′-CTCATATGCGCCTTGACT-3′	This study
bll7033 F/R	5′-CTGGTGTCGTTCTTCATGT-3′/5′-CTTCCACTTGTTGTACTCGTC-3′	This study

a Coordinate of the clones: brc02044, 7,723,981 to 7,747,611; brc00911, 7,735,146 to 7,758,873.

b Gene numbers are based on Rhizobase (http://www.kazusa.or.jp/rhizobase/) (21).

**Table 2:** Number, weight and acetylene reduction activity (ARA) of nodules formed with the combination of *Glycine max* (L.) cv. Enrei and deletion mutants or Wild-type of *B. japonicum* USDA110.

Inoculated strain	Nodule number [A] (number/plant)	Nodule dry weight [B] (mg/plant)	[B]/[A] (mg/nodule)	Acetylene reduction activity (ARA)	

				μmol/h/plant	nmol/h/nodule
Wild-type	5.70±0.82 (a)	6.25±0.42 (a)	1.10±0.09 (a)	0.29±0.08 (a)	50.91±6.43 (a)
ΔRND mutant	8.00±1.05 (b)	4.72±0.74 (b)	0.59±0.02 (b)	0.25±0.03 (a)	30.99±0.83 (b)
ΔPHB mutant	5.14±0.82 (a)	4.68±0.31 (b)	0.91±0.09 (a)	0.25±0.05 (a)	48.61±2.11 (a)

Values are the means ±S.D. of at least two replicate tests.

In each column, means not followed by the same letter differ significantly at the 5% level according to Student’s t-test.
